# Synthesis, reactivity and application studies for different biolubricants

**DOI:** 10.1186/1752-153X-8-16

**Published:** 2014-03-10

**Authors:** Jumat Salimon, Bashar Mudhaffar Abdullah, Rahimi M Yusop, Nadia Salih

**Affiliations:** 1 School of Chemical Sciences & Food Technology, Faculty of Science and Technology, Universiti Kebangsaan Malaysia, 43600 Bangi, Selangor, Malaysia

## Abstract

Vegetable oils have different unique properties owing to their unique chemical structure. Vegetable oils have a greater ability to lubricate and have higher viscosity indices. Therefore, they are being more closely examined as base oil for biolubricants and functional fluids. In spite of their many advantages, vegetable oils suffer from two major drawbacks of inadequate oxidative stability and poor low-temperature properties, which hinder their utilization as biolubricant base oils. Transforming alkene groups in fatty acids to other stable functional groups could improve the oxidative stability, whereas reducing structural uniformity of the oil by attaching alkyl side chains could improve the low-temperature performance. In that light, the epoxidation of unsaturated fatty acids is very interesting as it can provide diverse side chains arising from the mono- or di-epoxidation of the unsaturated fatty acid. Oxirane ring opening by an acid-catalyzed reaction with a suitable reagent provides interesting polyfunctional compounds.

## Introduction

Oils and fats have recently been used in environment friendly processes to produce tailor-made products and in novel chemical reactions to functionalize the carbon chain for the synthesis of new compounds. Most of the current developments are in the areas of green chemistry, cleaner energy-saving processes, renewable resources, and enzyme-catalyzed reactions [1]. Changing perceptions of what is nutritionally desirable in fat-based products also drives changing technology. Esterification is more widely used in the formulation of some modified fats [2].

Commercial oils and fats are mixtures of lipids. They are mainly triacylglycerols associated with diacylglycerols, monoacylglycerols, and free fatty acids in varying percentages. They may also contain phospholipids, free sterols and sterol esters, tocols, triterpene alcohols, hydrocarbons, and fat-soluble vitamins. The above description refers to the composition of crude oils when first extracted. During refining, some of the minor components are removed, wholly or in part, and useful materials may be recovered [3]. Each fat and oil molecule has a set of unique physical, chemical, and compositional parameters by which it can be recognized [4]. Traditional physical properties include density, melting behavior, refractive index, and viscosity [3]. The chemical properties include iodine value, saponification value or saponification equivalent, acetyl value, acid value, and peroxide value. These properties depend on their fatty acid content.

## Review

### Chemical structure of mineral and synthetic oils

Fatty acids, the main constituent of oils and fats, esterified using glycerol. The industrial exploitation of oils and fats for both food and oleochemical products is based on the chemical modification of both the carboxyl and unsaturated groups present in fatty acids. Although the most reactive sites in fatty acids are the carboxyl groups and the double bonds, the methylene groups adjacent to these functional groups are also activated, increasing the reactivity of the fatty acid. Only rarely do saturated chains show reactivity [1]. Carboxyl groups and unsaturated centers usually react independently; however, when in close proximity, both may react through neighboring group participation. In enzymatic reactions, the reactivity of the carboxyl group can be influenced by the presence of a proximate double bond [5].

Fatty acids from various sources exhibit many common features. This is a consequence of similarities in the biosynthesis of fats and oils throughout the vegetable and animal kingdoms. Small variations result from changes in the synthesizing enzymes, which, in some cases, can now be explained in terms of their amino-acid sequence. The following generalizations are true for most common fatty acids and for many of the minor acids described below although there are exceptions, which are sometimes significant [3]:

1. Natural fatty acids, both saturated and unsaturated, are straight-chain compounds with an even number of carbon atoms.

2. Unsaturated acids are often olefinic and have the *cis* configuration.

3. Polyunsaturated acids generally have a methylene-interrupted arrangement of *cis*-olefinic double bonds, such as that observed in linoleic acid. This pattern of unsaturation is characteristic of fatty acids, and is the cause for the characteristic reaction behaviour of fatty acids observed with oxygen, hydrogen, and alkalis.

4. Fatty acids rarely have functional groups other than the carboxyl group and the olefinic centers. Nevertheless, the fatty acids are categorized according to the following groups: hydroxy, epoxy, keto, or halogen.

Mineral oils, however, are extremely complex mixtures of C_20_-C_50_ hydrocarbons containing a range of linear alkanes (waxes), branched alkanes (paraffinics), alicyclic (naphthenics), olefinic, and aromatic species. They also contain significant amounts of heteroatoms, mainly sulfur. Mineral oils are more stable, cheaper, and more readily available than natural oils, and are also available in a wider range of viscosities. However, one issue regarding mineral oils is that oils derived from different sources have different characteristics. Another issue regarding mineral oils is the volatilization of low-molecular-weight components, which leads to a tendency to thicken during use. The presence of low-molecular-weight components also reduces the flash point of mineral oils compared to natural oils of the same viscosity [6]. There are a range of synthetic lubricants such as polyolefins (PAOs) that have characteristics similar to highly refined paraffinic mineral oils, but with a narrower molecular weight distribution. Alkyl benzenes are a class of synthetic hydrocarbons; the lubricant industry, however, almost exclusively uses branched alkyl benzenes as they have better low-temperature fluidity [7].

Esters were originally developed for the lubrication of aircraft jet engines [8] but have subsequently found widespread use, particularly in applications where biodegradability is required. The presence of the ester group in lubricants confers low-temperature fluidity and reduces volatility at high temperatures. It also provides an affinity for metal surfaces [8]. For applications where chemical stability is an overriding requirement, non-hydrocarbon-based fluids such as poly(dimethyl siloxanes) and perfluoroalkyl ethers may be used. However, the use of these non-hydrocarbon-based biolubricants is restricted by their relatively high cost and their incompatibility with other biolubricants and standard additives. All synthetic biolubricants are normally used as formulations containing the same types of functional additives as are used in mineral oils [9]. Table 1 shows further information regarding the synthetic lubricants available in the market.

**Table 1:** **Type and application of synthetic biolubricants **[9]

**No.**	**Class**	**Type**	**Operating temperature °C**	**Applications**	**Advantages vs. mineral oil**	**Limiting properties**
1.	Synthesized fluids hydrocarbons (SFHs)	Polyalphaolefins, alkylated aromatics, polybutenes, cycloaliphatic	155 to -45	Machine tool spindles, Freezer plants-motors, conveyors, bearings	High temperature stability, long life, low temperature fluidity, high viscosity index, improved wear protection, low volatility, oil economy	Solvency/detergency, seal compatibility
2.	Organic esters	Dibasic acid ester, polyol ester	204 to -35	Commercial manual transmission	No wax, high temperature stability, long life, low temperature fluidity, solvency/detergency	Seal compatibility, mineral oil compatibility, antiwear and extreme pressure, hydrolytic stability, paint compatibility
3.	Phosphate esters (phosphoric acid esters)	Triaryl phosphate ester, trialkyl phosphate ester, mixed alkylaryl phosphate esters	180 to -18	Hydraulic Systems	Fiber resistance, lubricating ability	Seal compatibility, low viscosity index, paint compatibility, metal corrosion, hydrolytic stability
4.	Polyglycols	Polyalkylene, polyoxyalklylene, polyethers, glycols	245 to -20	Gas turbines	Water versatility, high viscosity index, low temperature fluidity, antirust, no wax	Mineral oil compatibility, paint compatibility, oxidation stability

Source: Salimon et al. [9].

### *Jatropha curcas* seed oil

*J. curcas* can grow well under adverse climatic conditions because of its low moisture demands, fertility requirements and tolerance to high temperatures [10]. It is easy to grow, grows relatively quickly, and is hardy (being tolerant to drought). However, it is not grown widely because its leaves and stems are toxic to animals; however, the seeds or seed cake can be used as an animal feed. Various parts of the plant are of medicinal value; its bark contains tannin, while its wood and fruit can be used for numerous purposes, including as fuel [11,12].

The latex produced from the branches is useful for wound healing and other medical uses. Each fruit contains 2-3 oblong black seeds, which can produce oil. The seed kernel contains 40-60 wt% of oil [13]. The extracted seed oil is useful for medicinal and veterinary purposes, as an insecticide, for soap production and as a fuel substrate [11]. The two principal objectives of studies such as these are to use oil plants and their products for economic and environmentally sustainable rural development and to make rural areas self-sufficient in energy, especially liquid fuels. Where possible, this is to be achieved without displacing other agricultural crops or competing for land that is more suitable for other applications. *J. curcas* was chosen as one of the prime plant oil species, especially from Brazil, Nepal, Malaysia and Zimbabwe. This study focuses on *J. curcas* seed oil because it is toxic and cannot be used for food consumption. Moreover, it has many industrial applications such as biodiesel, biolubricants, and polymers [12]. Studies on the physical and chemical characteristics of *J. curcas* seed oil are quite extensive.

A comparative evaluation of the physicochemical properties of *J. curcas* seed oil from Malaysia, Indonesia, and Thailand has been conducted previously [14]. Physicochemical properties such as the density, viscosity, fatty acid (FA)%, iodine value, saponification value, and peroxide value of the *J. curcas* seed oil were evaluated. The evaluation of fatty acid composition revealed that the oleic (42.4-48.8%) and linoleic acids (28.8-34.6%) were the dominant fatty acids present in *J. curcas* seed oil. Saturated fatty acids such as palmitic and stearic acid were found to be present in the range of 4.5–13.25% and 7–7.7%, respectively. The major triacylglycerols (TAG) were observed to be oleic-oleic-linoleic (OOL) 22.94–25.75% and oleic-linoleic-linoleic (OLL) 15.52–20.77%.

The kinetics of epoxidation of *J. curcas* seed oil by peroxyacetic or peroxyformic acid, formed *in situ* by the reaction of aqueous hydrogen peroxide and acetic/formic acid, in the presence of an acidic ion exchange resin as catalyst in or without toluene, was studied by [15]. The presence of an inert solvent in the reaction mixture appeared to stabilize the epoxidation product and minimize any side reaction, such as the opening of the oxirane ring. The effect of several reaction parameters such as stirring speed, hydrogen peroxide-to-ethylenic unsaturation molar ratio, acetic/formic acid-to-ethylenic unsaturation molar ratio, temperature, and catalyst loading on the epoxidation rate as well as on the oxirane ring stability and iodine value of the epoxidized *J. curcas* oil were examined. The multiphase process consisted of a consecutive reaction comprising an acidic ion exchange resin-catalyzed peroxyacid formation followed by epoxidation. The catalytic reaction of peroxyacetic/peroxyformic acid formation was found to be characterized by the adsorption of only acetic (or formic) acid and peroxyacetic/peroxyformic acid on the active catalyst sites, and the irreversible surface reaction was the overall rate determining step. The proposed kinetic model takes into consideration two side reactions, namely, epoxy ring opening involving the formation of hydroxyl acetate and hydroxyl groups and the reaction of the peroxyacid and epoxy group. The kinetic and adsorption constants of the rate equations were estimated by the best fit using the nonlinear regression method. Good agreement between experimental and predicted data validated the proposed kinetic model. Small values of kinetic rate constants for both the side reactions indicated that the ring-opening reactions were relatively much slower. The activation energy for the epoxidation reaction was determined to be 53.6 kJ/mol.

*J. curcas* seed oil and soybean oil have high a content of unsaturated fatty acids that can be converted to epoxy fatty acids, as reported by [16]. The plant oil-based epoxies are sustainable, renewable, and biodegradable materials that can replace petrochemical-based epoxy materials in some applications. To produce epoxidized soybean oil and epoxidized *J. curcas* seed oil, we carried out the epoxidation reaction using conventional chemistry at 50°C under atmospheric pressure for about 10 h. The maximum reaction conversion was 83.3% for the epoxidation of soybean oil and 87.4% for the epoxidation of *J. curcas* seed oil. The presence of an excess amount of hydrogen peroxide was necessary in the reaction to achieve high reaction conversion. The highest epoxy content observed in epoxidized soybean oil was 6.13 wt%, which is comparable to the epoxy content in commercially available epoxidized soybean oil. The highest epoxy content observed in epoxidized *J. curcas* seed oil was 4.75 wt%; unfortunately, there is no commercially available epoxidized *J. curcas* seed oil to make a comparison. It is possible to produce epoxidized *J. curcas* seed oil in Thailand as *J. curcas* seed oil is produced locally unlike soybean oil, which is imported. However, the applications of epoxidized *J. curcas* seed oil from Thailand are limited owing to its lower epoxy content, despite the oil extracts exhibiting good physicochemical properties. In contrast, the fatty acids of Malaysian *J. curcas* seed oil have a great potential in oleochemical applications such as surface coatings, biodiesels, and biolubricants. Therefore, it is pragmatic to carry out more research on Malaysian *J. curcas* seed oil and its fatty acids in the future to explore its potential for future industrial oilseed crops.

### Biolubricants

**Figure 1:**
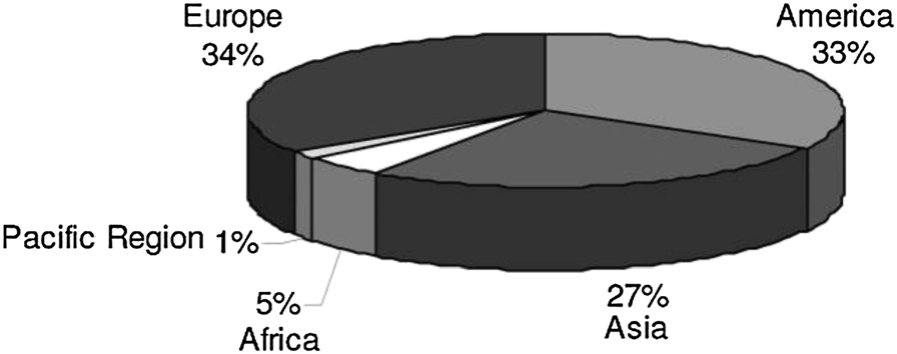
**Worldwide biolubricant market **[9]**.**

The many terms that are used for the classification of biolubricants and their products include environmentally friendly, environmentally acceptable, biodegradable, and non-toxic. Approximately 1% of the total mineral oil consumed is used to formulate biolubricants [9]. Figure 1 reveals the worldwide market for biolubricants with altered physical properties and appearances [9]. Biolubricants that remain in the environment also include those used in circulation systems, which are not collected or disposed of. In addition, leaked biolubricants and those remaining in filters or containers have to be taken into account. Based on the above-mentioned statistic, it was determined that the environment in Germany is exposed to about 150,000 tons of biolubricants annually, which represents the volume of biolubricant that returns to the environment [17]. A calculation based on the actual biolubricant consumption in Germany and the disposal rates for different types of biolubricants reveal that the total volume of biolubricants could be about 250,000 tons annually. Consideration of the volume representing lost biolubricants and undefined biolubricants accounted for the total volume of biolubricants in Germany. It is likely that the volume of biolubricants returning to the environment may be in the order of at least 300 000 t/a [18].

The production, application, and disposal of biolubricants have to meet safety guidelines in order to provide the best possible protection for the environment and living beings in particular. Most often, health hazards to humans are derived from indirect routes through the environment. For all cases of direct contact between biolubricants and human beings, compatibility has to be verified. All measures have to be taken to minimize damage to the environment. In evaluating the seriousness of the detrimental effects on the environment, the advantages of biolubricants, such as their performance or economic properties, must be considered and weighed against the risks associated with these biolubricants [19]. The term biolubricant applies to all biolubricants that are both rapidly biodegradable and non-toxic to humans and aquatic environments. A biolubricant may be vegetable oil-based such as rapeseed oil or derived from synthetic esters manufactured from modified renewable oils or from mineral oil-based products [20].

Typically, biolubricants designed for one application are not suitable for use in another application without a loss in performance. Equipment manufacturers play a predominant role in recommending viscosity grades and biolubricant quality based upon their system needs. Biolubricant properties that are commonly considered for assessing the suitability of a biolubricant for a particular application include the fluidity range, viscosity index, low-temperature fluidity, oxidation stability (inhibited), hydrolytic stability, thermal stability, mineral oil compatibility, additive solvency, volatility, rust control (inhibited), boundary lubrication, fire resistance, elastomer compatibility (especially with buna rubber), and relative cost [21]. Additional properties that are important in some other applications include color, density, volatility, bulk modulus, shear stability, acidity and alkalinity, detergency, and foaming and air release tendency [21]. Current research efforts are directed towards improving the low-temperature stability of vegetable oils by chemical modification, blending with functional fluids, and by the use of additives. The main properties to take into account are as follows:

a. Pour Point

For proper lubrication of mechanical equipment, petroleum-based biolubricants that operate in the liquid phase have been designed. Accordingly, for a base stock, it is important to know the temperature at which the transition between the liquid and solid phases occurs and manufacture it to be as low as necessary. This temperature is known as the pour point (PP) and is defined as the lowest temperature at which movement of the specimen is observed [22]. PP values have traditionally been measured when the sample no longer moves on tilting the tube containing the sample (pour point). Pour points lower than 0˚C are considered to be low [3].

b. Flash Point

The flash point is defined as the minimum temperature at which a liquid produces a sufficient concentration of vapor above it to form an ignitable mixture with air. Oils with a lower flash point are a greater fire hazard. The flash point should be high enough to allow safe operation and minimum volatilization at the maximum operating temperature. For the most demanding applications, such as aviation jet engine biolubricants, an effective liquid range over 300°C may be required [9].

c. Viscosity

The viscosity of fatty acids and vegetable oils is a quantitative measure of its resistance to flow. It is the key property of base stocks since it is a major factor in determining their application; for example, low viscosity stocks can be used for automotive transmission oils, while higher viscosity stocks are employed in diesel engine oils. Base stocks are usually named according to their viscosity. Viscosity measurements on base stocks assume that the liquids are Newtonian in which shear stress and shear rate are linearly related [22]. Viscosity is critical to determining the quality of a biolubricant film. In metal forming applications, the biolubricant viscosity determines the effectiveness of the film in separating the tool from the work-piece, thereby controlling friction and wear. Metal removal operations, on the other hand, have diverse lubrication needs, and, hence, the optimum biolubricant viscosity must be estimated for each operation. This is accomplished by considering the ability of the biolubricant to enter and remain in the contact zone, the durability of the biolubricant film, the desired rate of spreading, and its cooling capability [21].

d. Oxidative Stability

Oxidation is the most important reaction of oils resulting in increased acidity, corrosion, viscosity, and volatility when biolubricant-based oils are used as engine oils. The triacylglycerol structure forms the backbone of most available vegetable oils that comprise different fatty acid chains. Therefore, a complex association of different fatty acid molecules attached to a single triacylglycerol structure constitutes a vegetable oil matrix. The presence of unsaturation in the triacylglycerol molecule, owing to the presence of oleic, linoleic, and linolenic acid moieties, functions as the active site for various oxidation reactions. Saturated fatty acids have relatively high oxidation stability, which decreases with increasing unsaturation in the molecule [23]. Several oxidation tests are available primarily as screening tools for oxidative stability of fatty acids and vegetable oils. The evaluation of oxidation is extremely complex, and a fully acceptable protocol is yet to emerge [24].

The direct use of vegetable oils as biolubricants has disadvantages because of a variety of factors. Vegetable oils have poor oxidative and thermal stability due to the presence of acyl groups. The presence of the glycerol backbone in oil gives rise to a tertiary β-hydrogen, which is thermally unstable. Fortunately, there are different ways and methods to overcome it. For example, the chemical modification of vegetable oils by reactions such as epoxidation, esterification, and acetylation across the double bonds constitutes a promising method for obtaining valuable commercial products from renewable raw materials [9].

In order to use vegetable oil with special additives such as antioxidants, viscosity modifiers, rust inhibitors, wear reducers, pour point depressors (PPD), de-emulsifiers, and hydrolysis inhibitors to improve biolubricant properties, chemical modifications, de novo synthesis, breeding and biotechnology play an important role. These methods improve the performance and stability of base oils in lubricating formulations. They also allow the use of vegetable-based oil substrates for green engineering [9].

Chemical modifications such as epoxidation, oxirane ring opening, and esterification of vegetable oils have been shown to improve the PP, flash point, viscosity and oxidative stability of the vegetable oil-based biolubricants and to achieve optimal characteristics for extreme applications [25].

#### ***Epoxidation reaction***

**Figure 2:**
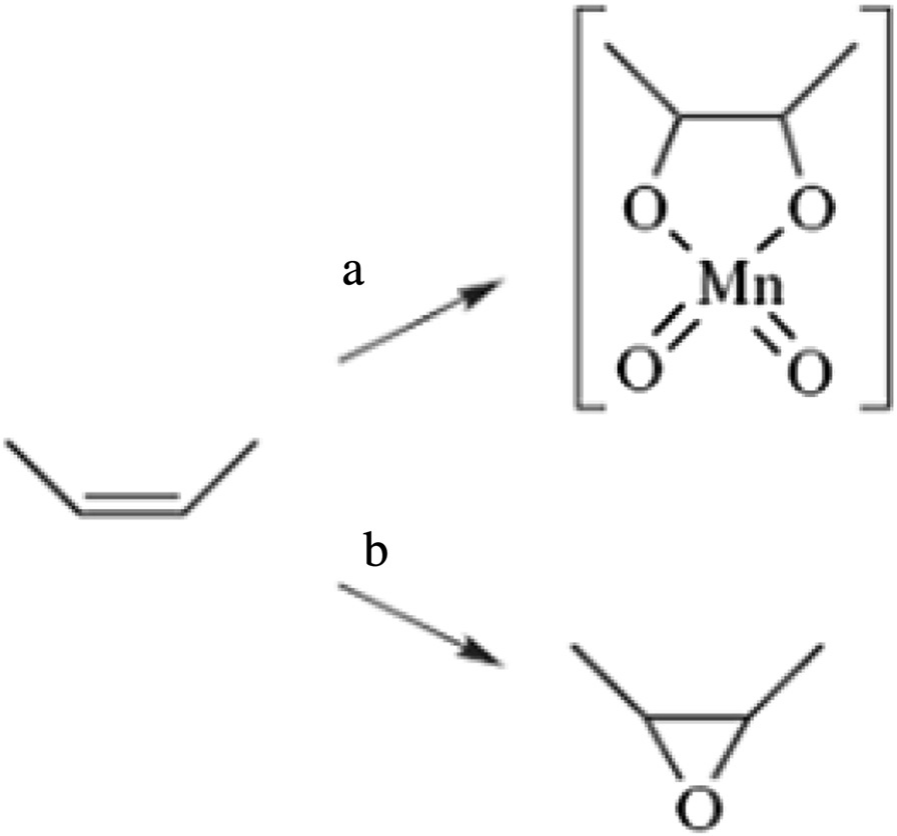
**Stereochemistry of epoxidation reactions with KMnO **_**4 **_**(a); m-chloroperbenzoic acid (b) catalyzed hydrolysis **[26]**.**

Epoxides are produced by the reaction of double bonds with peracids. This proceeds by a concerted mechanism, giving *cis* stereospecific addition. Thus, a *cis* olefin leads to a *cis* epoxide and a trans-olefin to a trans-epoxide. The carboxylic acid produced is a stronger acid than the strongly hydrogen bonded peracid and may lead to subsequent ring opening reactions, especially in the case of formic acid. Small-scale reactions are carried out using m-chloroperbenzoic acid in a halocarbon or aromatic solvent in the presence of bicarbonate to neutralize the carboxylic acid as soon as it is formed [26]. Epoxides are highly reactive and readily undergo ring-opening reactions in acid following protonation of the epoxy oxygen (Figure 2). This is a route commonly employed for the synthesis of diols and polyols that are used in the production of polymers and a range of hydroxyl compounds. Ring opening of methylene-interrupted di-epoxides leads to the formation of 5- and 6-membered ring ethers through neighboring group participation [26]. Many studies have indicated the importance of using catalysts for epoxidation purposes.

The epoxidation of Mahua oil (*Madhumica indica*) by hydrogen peroxide was studied by [27]. Mahua oil with an iodine value of 88 g/100 g, and containing 46% oleic acid and 12.74% linoleic acid, was epoxidized *in situ* with hydrogen peroxide as an oxygen donor and glacial acetic acid as an active oxygen carrier in the presence of a catalytic amount of an inorganic acid. Higher temperatures and higher sulfuric acid concentrations reduced the reaction time and resulted in higher oxirane content with less cleavage to glycol. H_2_SO_4_ was found to be more effective in terms of oxirane conversion. The epoxidation reaction of mahua oil fell into a kinetically controlled regime at stirring speeds >1500 rev/min. From the relative conversion data obtained for various reaction parameters, it can be concluded that it is possible to develop value added products, such as epoxides, from Mahua oil.

A study of the epoxidation of soybean oil and soybean methyl esters with a dilute solution of hydrogen peroxide (6 wt%) using an amorphous heterogeneous Ti/SiO_2_ catalyst in the presence of tert-butyl alcohol was studied by [28]. The highest yields of epoxidized olefins were obtained on using a H_2_O_2_: substrate molar ratio of 1:1. Ratios higher than this were not effective in speeding up the reaction. The parameters affecting the lipase activity and operational lifetime of the chemo-enzymatic epoxidation of fatty acids (oleic acid, palmitic acid, and epoxystearic acid) by immobilized *Candida antarctica* lipase B (Novozym^®^ 435) has been studied previously [29]. In the presence of 6-12 M hydrogen peroxide, the enzyme was rather stable at 20°C, while at 60°C, the enzyme lost activity rapidly, with the rate of deactivation increasing with hydrogen peroxide concentration. In the work presented here, the parameters found to be most crucial for the activity and, hence, the operational stability of Novozym^®^ 435 in the chemo-enzymatic epoxidation of fatty acids, were the high concentration of hydrogen peroxide and elevated temperatures. For epoxidation processes run at elevated temperatures, the controlled addition of H_2_O_2_ is important for enzyme stability, more so in the initial stages of the reaction, where the formation of water is sufficient to dilute the added H_2_O_2_. Since the reaction is exothermic, a large-scale process would probably be most efficient if a temperature program is used. Concurrent with improving the process design, development of a more stable biocatalyst preparation would be an alternative strategy.

Canola oil, with an iodine value of 112/100 g, and containing 60% oleic acid and 20% linoleic acid, was epoxidized using a peroxyacid generated *in situ* from hydrogen peroxide and a carboxylic acid (acetic or formic acid) in the presence of an acidic ion exchange resin (AIER), Amberlite IR 120H [30]. Acetic acid was found to be a better oxygen carrier than formic acid, as it resulted in about 10% more conversion of the ethylenic unsaturation to oxirane than that produced by formic acid under otherwise identical conditions. The parameters optimized were temperature (65°C), acetic acid to ethylenic unsaturation molar ratio (0.5), hydrogen peroxide to ethylenic unsaturation molar ratio (1.5), and AIER loading (22%). An iodine conversion of 88.4% and a relative conversion to oxirane of 90% were obtained under the optimum reaction conditions. The heterogeneous catalyst, AIER, was found to be reusable and exhibited a negligible loss in activity.

Another study attempted to show the efficiency of epoxidation under different catalysts conditions [31]. It was found that different catalysts have been used for the epoxidation of oil and unsaturated fatty acids with almost complete conversion to form the monoepoxide of the unsaturated carbon. Catalysts such as H_2_SO_4_, Ti(IV)-grafted silica catalysts, tungsten-based catalysts, acidic ion-exchange resins, potassium peroxomonosulfate, and alumina catalysts have been studied. H_2_SO_4_ was found to be more effective in terms of the complete conversion of oxirane. The epoxidation of oils and unsaturated fatty acids is widely used for the production of oxiranes, which are valuable industrial products that provide an access to various important chemicals; unfortunately, none of them have found an industrial application as yet.

The effect of reaction parameters on the lipase-mediated chemo-enzymatic monoepoxidation of linoleic acid was investigated by [32]. Hydrogen peroxide was found to have the most significant effect on the reaction rate and degree of epoxidation. An excess of hydrogen peroxide with respect to the unsaturation was necessary in order to yield total conversion within a short time period, as well as to compensate for hydrogen peroxide decomposition at temperatures above 50°C. The reaction rate also increased with hydrogen peroxide concentration (between 10 and 50 wt%), albeit at the expense of enzyme inactivation. Linoleic acid was completely epoxidized when used at a concentration of 0.5-2 M in toluene at 30°C, while in a solvent-free medium, the reaction was not complete due to the formation of a solid or a highly viscous oily phase, creating mass transfer limitations. Increasing the temperature up to 60°C also improved the rate of epoxide formation.

The monoepoxidation of methyl linoleate was examined using transition metal complexes as catalysts [33]. With a catalytic amount of methyltrioxorhenium (MTO) 4 mol% and pyridine, methyl linoleate was completely epoxidized by aqueous H_2_O_2_ within 4 h. Longer reaction times (6 h) were needed with 1 mol% catalyst loading. Manganese tetraphenylporphyrin chloride was found to catalyze the partial epoxidation of methyl linoleate. A monoepoxidized species was obtained as the major product (63%) after 20 h.

Linoleic acid (LA) is converted to per-carboxylic acid, catalyzed by an immobilized lipase from *Candida antarctica* (Novozym 435) [34]. This per-carboxylic acid is only an intermediate, which undergoes self-epoxidation in good yields and almost without consecutive reactions. Reactions conditions for the monoepoxide linoleic acid 9(12)-10(13)-monoepoxy 12(9)-octadecanoic acid (MEOA) was optimized using D-optimal design. Under optimum conditions, higher yields (82.14%) and medium oxirane oxygen content (OOC) (4.91%) of MEOA were predicted at 15 *μ*L of H2O2, 120 mg of Novozym 435, and 7 h of reaction time. In order to develop better quality biolubricants, we determined the pour point (PP), flash point (FP), viscosity index (VI), and oxidative stability (OT) for LA and MEOA. The results showed that MEOA exhibited good low-temperature behavior with a PP of *-*41°C. The FP of MEOA increased to 128°C comparing with 115°C of LA. Similarly, the VI for LA was 224, several hundred centistokes (cSt) more viscous than MEOA, which had a VI of 130.8. The ability to resist oxidative degradation is another important property for biolubricants. Therefore, LA and MEOA were screened to measure their OT, which was observed at 189 and 168°C, respectively.

Recent studies have attempted to improve the efficiency of monoepoxidation under milder conditions by minimizing the formation of by-products. The lipase is remarkably stable under the reaction conditions and can be recovered and reused 15 times without loss of activity.

#### ***Oxirane ring-opening reaction***

Because of the high reactivity of the oxirane ring, the epoxidation of the double bonds opens up a wide range of reactions that can be carried out under moderate reaction conditions. A variety of chemical modifications of epoxidized vegetable oils and fatty acids are possible through epoxy moiety, and one of the most commonly used is the ring-opening reaction [35].

**Figure 3:**
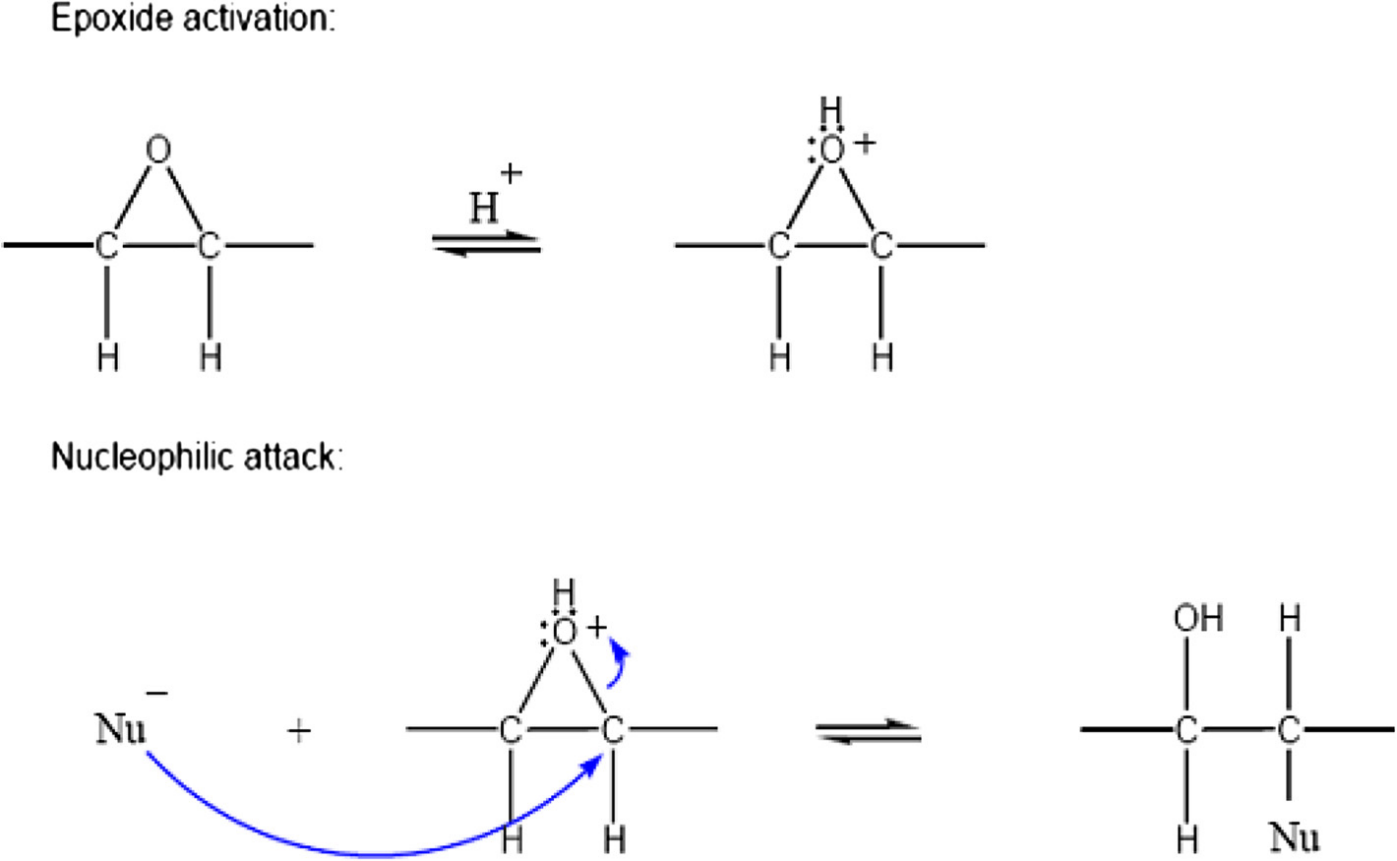
**Acid catalyzed nucleophilic attack on an epoxide **[36]**.**

Ring opening takes place through cleavage of one of the carbon-oxygen bonds. It can be initiated by either electrophiles or nucleophiles, or catalyzed by either acids or bases. For example, the acid-catalyzed hydrolysis of an epoxide is a useful procedure for preparing vicinal-dihydroxy compounds (glycols) [36]. The nucleophilic addition of a carboxyl group to the epoxide center can easily be promoted by protonation using solid acid catalysts (Figure 3). Nonetheless, the rate of the oxirane ring opening of epoxidized fatty acids strongly depends on the nature and structure of the carboxylic acid [37].

Acid catalysis assists epoxide ring opening by providing a better leaving group (an alcohol) at the carbon undergoing nucleophilic attack. This catalysis is especially important if the nucleophile is a weak one such as water or an alcohol. In the absence of an acid catalyst the leaving group must be a strongly basic alkoxide ion; however, such a reaction is not very environmentally friendly. Although such reactions do not occur with other ethers, they are possible with epoxides, provided the attacking nucleophile is strong enough [36]. There have been many studies on oxirane ring opening using different alcohols, catalysts, and carboxylic acids, as will be described in the following few paragraphs.

9,12-hydroxy-10,13-oleioxy-12-octadecanoic acid was synthesized based on the esterification reaction of monoepoxide linoleic acid 9(12)-10(13)-monoepoxy 12(9)-octadecanoic acid (MEOA) with oleic acid (OA) and catalyzed by *p*-toluenesulfonic acid (PTSA) [38,39]. The optimum conditions for the experiment using D-optimal design to obtain high yield% of 84.61, conversion% of 83.54 and lowest OOC% of 0.05 were predicted at an OA/MEOA ratio of 0.2:1 (mol/mol), PTSA/MEOA ratio of 0.4:1 (mol/mol), reaction temperature of 110°C, and reaction time of 4.5 h. The results showed that an increase in the chain length of the mid-chain ester resulted in a decrease of the pour point to -51°C, an increase in the viscosity index up to 153, and an improvement in the oxidative stability to 180.94°C.

The degradation of the oxirane ring of epoxidized vegetable oils with hydrogen peroxide using an ion exchange resin (Amberlite IR-120) was done in a previous study [40]. The results showed that the ring opening increases either by adding a higher amount of catalyst to the system or by decreasing the particle size of the catalyst since, in both cases, the external area of the catalyst becomes larger. Despite the unavoidable presence of surface-exposed protons, the degradation can be reduced by several orders of magnitude for similar process conditions by using such a heterogeneous catalyst instead of mineral acids. Although the use of such ion exchange resins does not completely eliminate the attack on the oxirane group by H_2_O_2_ during the epoxidation process, this deleterious reaction only seems to be important under extreme process conditions, such as high temperature, excessive concentration of H_2_O_2_, and high molar ratio of H_2_O_2_ to double bonds, which are not common in industrial practice.

Another study focused on the main oxirane ring-opening reactions that occur during the manufacture of epoxidized vegetable oils using a strongly acidic, gel-type ion exchange resin (IER) (Amberlite IR-120, 8% cross linking) [41]. The combined results on the attack on the oxirane ring of epoxidized vegetable oils by either H_2_O_2_ or solvated acetic acid indicate that under process conditions, these attacks proceed in the kinetic regime; that is, they are not mass-transfer controlled. These results indicate that most of the degradation occurs on the catalyst and confirm that the external surface protons of the IER are mainly responsible for the deleterious degradation of the oxirane ring, since, in both cases, the degradation rate was directly proportional to the available external area of the catalyst.

The ring-opening reaction of epoxidized soybean oil with different alcohols such as *n*-butanol, *iso*-amyl alcohol and 2-ethylhexanol was carried out in the presence of Amberlyst 15 (Dry) as a catalyst [42]. The observed pour points of the products were in the range of -5 to -15°C. The hydroxy group of the ring-opening product of *n*-butanol further reacted with acetic anhydride in the presence of Amberlyst 15 (Dry), which was previously used to carry out ring-opening reaction by alcoholysis. Oxirane ring opening was achieved using different alcohols and different carboxylic acids. Thus, these studies show that the high cleavage of the oxirane ring depends on the type of catalyst and on temperature.

#### ***Esterification reaction***

Fatty acids are converted to esters through a reaction with excess of alcohol using an acid catalyst or lipase. Boron trifluoride, sulfuric acid, or anhydrous hydrogen chloride in methanol are commonly used for preparing methyl esters [43]. The reaction is completed in 30 minutes at reflux. Propyl and butyl esters are prepared similarly with the corresponding alcohols. Excess alcohol cannot always be used, for example, in the synthesis of triacylglycerols using a protected glycerol. A more reactive fatty acid derivative such as the acid chloride or anhydride is used, or the fatty acid reacts directly with the alcohol, using dicyclohexylcarbodiimide and 4-dimethylaminopyridine as a coupling agent, for example, in the synthesis of acylglycerols. Some groups in more uncommon fatty acids are acid sensitive, for example, epoxides, cyclopropanes, cyclopropenes, and hydroxy compounds, and hence, methods that do not involve acids catalysts are needed. Reaction with diazomethane or the less-hazardous trimethylsilyl-diazomethane is possible [43]. Many studies have been carried out on the esterification process using different catalysts and alcohols.

A previous study focused on the esterification reaction of fusel oil and oleic acid, in which immobilised Novozym 435^®^ lipase enzyme was used as a biocatalyst [44]. Compared to the product obtained by acid catalysis, in the esterification reaction, there was no trace of oleic acid since the entire conversion was achieved by continuous water removal through evaporation. The results showed that the method could achieve 99.8% conversion under optimal conditions. The oleochemical ester produced does not have aquatic toxicity and the determined tribological and physicochemical properties of the biolubricant proved that it is an environmentally friendly product.

Several diesters have been prepared from commercially available oleic acid and common organic acids [45]. The key step in the three-step synthesis of oleochemical diesters involves the ring-opening esterification of alkyl 9,10-epoxyoctadecanoates, such as propyl, isopropyl, octyl, and 2-ethylhexyl, using propionic and octanoic acids without the need for solvents or catalysts. The increasing chain length of the mid-chain ester and branching in the end-chain ester was found to have a positive influence on the low-temperature properties of the diester. Improved oxidation stability is achieved when the chain length of the mid-chain ester is increased. Additionally, the mid-chain ester plays a larger role in oxidation stability than the end-chain ester. These products may prove useful in the search for bio-based industrial materials, such as biolubricants, surfactants, and fuel additives.

### Polyester synthesis

**Figure 4:**
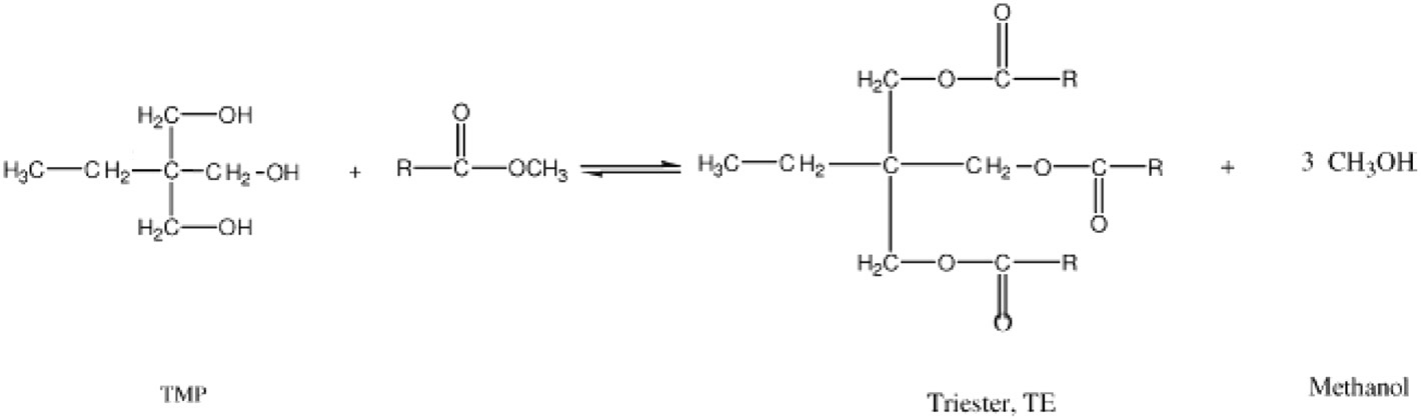
**Trimethylolpropan esterification **[46]**.**

Biodegradable organic polyesters derived from the esterification of vegetable oils and branched neopolyols such as trimethylolpropan (TMP) and PE have been developed for various applications (Figure 4). In a previous study, biodegradable TMP 2-ethyl- 2-hydroxymethyl-1,3-propanediol esters of rapeseed oil fatty acids were synthesized using enzymatic and chemical methods [46]. Sodium methylate (0.5% w/w) was employed as a catalyst, and the reaction mixture was refluxed under a reduced pressure of 3.3 kPa. Approximately 99% of conversion was achieved at 110–120°C in 10 h. By using 40% w/w *Candida rugosa* lipase, only 64% of the TMP was converted to triester in 24 h at 5.3 kPa at 47°C. With immobilized *Rhizomucor miehei* (50% w/w), the highest conversion to the TMP triester was 90% in 66 h.

Another study demonstrated that palm oil TMP esters containing 98% w/w triester can be successfully synthesized in less than an hour [47]. The chemical transesterification of TMP with palm oil methyl esters was achieved under a reduced pressure of at least 20 mbar at 120°C with a molar ratio 3.9:1 using sodium methoxide as a catalyst. The optimum molar ratio was established as 3.9:1, and less than 1.0% w/w of the catalyst was required, which is much lower than the lipase required for enzymatic transesterification (40–50% w/w) [48].

Esters of neopentylpolyols have also been prepared by an esterification reaction between PE and erucic acid catalyzed by PTSA in xylenes [49]. The reaction mixture was heated to 200°C under a nitrogen atmosphere. Since PE forms the backbone of the new esters, four types of esters were obtained, including tetra-, tri-, di-, and monoesters. These esters provided improved low-temperature behaviour. Animal fats have also been used to synthesize polyol esters using calcium methoxide; however, the rate of the reaction was slow. The yield of the reaction was 85–90% after 20 h [50]. A two-stage low-temperature crystallization process was used to improve the PP.

Another study demonstrated the use of functionalization to overcome these disadvantages [51]. In this work, mono-, tri- and tetra-esters were synthesized, including 10,12-dihydroxy-9 (stearoyloxy) octadecanoic acid 3; 9,10,12-tris(stearoyloxy)octadecanoic acid 4; and 18-(4-ethylhexyloxy)-18-oxooctadecane-7,9,10- triyl tristearate 5. Pour-point and cloud-point measurements showed that these derivatives have improved low-temperature properties as compared to the precursor. The tetraester compound, 18-(4-ethylhexyloxy)-18-oxooctadecane-7,9,10-triyl tristearate 5, had the lowest pour point of -44.37°C and the lowest cloud point of -41.25°C. This derivatization also improved the compound’s thermo-oxidative stability, measured using pressurized differential scanning calorimetry (PDSC) and thin-film micro-oxidation (TFMO) testing. 18-(4-ethylhexyloxy)-18-oxooctadecane-7,9,10-triyl tristearate 5 also had the highest onset temperature (OT) (282.10°C) and the lowest volatile loss and insoluble deposit (37.39% and 50.87%, respectively). Furthermore, the tribological behaviors of the compounds were evaluated using the four-ball method. 18-(4-ethylhexyloxy)-18-oxooctadecane-7,9,10-triyl tristearate 5 also had the lowest coefficient of friction (μ) (0.44). The results showed that, in general, these derivatives exhibit good anti-wear and friction-reducing properties at relatively low concentrations, under all considered test loads.

Oleyl 9(12)-hydroxy-10(13)-oleioxy-12(9)-octadecanoate has been synthesized based on the esterification reaction of 9,12-hydroxy-10,13-oleioxy-12-octadecanoic acid with oleyl alcohol and catalyzed by sulfuric acid [52,53]. The following were considered optimum conditions to obtain high yield%: OL/HYOOA ratio of 2:1 mol/mol, SA/HYOOA ratio of 0.7:1 mol/mol, reaction temperature of 110°C, and reaction time of 7 h. Under these conditions, the yield was 88.7%. The physicochemical characteristics were also determined, which showed improved low-temperature (-62°C) properties, viscosity index of 192, and increased oxidative stability up to 215°C.

## Conclusions

Vegetable bio-based oils are an important part of developing new strategies, policies, and subsidies that aid in reducing the dependence on mineral oil and other non-renewable sources. Vegetable oils exhibit unique chemical structures, and hence, their properties differ from those of mineral oils. By chemically modifying vegetable oils (through reactions such as epoxidation, esterification, and acetylation), their characteristics such as sensitivity to hydrolysis and oxidative attacks, low-temperature properties, and viscosity index coefficients can be improved. Chemically modified vegetable oils exhibit better lubrication ability, viscosity indices, and superior anticorrosion properties, because of the improved affinity of vegetable oils to metal surfaces. Vegetable oils can be used in an extremely wide range of automotive and industrial applications. Nonetheless, identifying a biolubricant that is both cost-effective and environment-friendly would be a challenge.

## Competing interests

The authors declare that they have no competing interests.

## Authors’ contributions

BMA & JS developed the concept analyzed the data and drafted the manuscript. RMY provided advice on the testing methods. NS performed the characterization methods. All of the authors read and approved the final manuscript.
